# ESR Essentials: Imaging in colorectal cancer—practice recommendations by ESGAR

**DOI:** 10.1007/s00330-024-10645-3

**Published:** 2024-02-28

**Authors:** Damiano Caruso, Michela Polici, Davide Bellini, Andrea Laghi

**Affiliations:** 1 Radiology Unit, Department of Medical Surgical Sciences and Translational Medicine, “Sapienza” University of Rome, Sant’Andrea University Hospital, Via Di Grottarossa, 1035-1039, 00189 Rome, Italy; 2 PhD School in Traslational Medicine and Oncology, Department of Medical Surgical Sciences and Translational Medicine, Faculty of Medicine and Psychology, “Sapienza” University of Rome, Rome, Italy; 3 Department of Radiological Sciences, Oncology and Pathology, “Sapienza” University of Rome, I.C.O.T. Hospital, Latina, Italy

**Keywords:** Colon cancer, Rectal cancer, Imaging, CT, MR

## Abstract

**Abstract:**

Colorectal cancer (CRC) is a significant global health concern. Diagnostic imaging, using different modalities, has a pivotal role in CRC, from early detection (i.e., screening) to follow-up. The role of imaging in CRC screening depends on each country’s approach: if an organized screening program is in place, the role of CT colonography (CTC) is limited to the study of either individuals with a positive stool test unwilling/unable to undergo colonoscopy (CC) or in patients with incomplete CC. Although CC is the most common modality to diagnose CRC, CRC can be also incidentally detected during a routine abdominal imaging examination or at the emergency room in patients presenting with intestinal occlusion/subocclusion or perforation. Staging is a crucial aspect of CRC management, guiding treatment decisions and providing valuable prognostic information. An accurate local staging is mandatory in both rectal and colon cancer to drive the appropriate therapeutic workflow. Important limitations of US, CT, and MR in N-staging can be partially solved by FDG PET/CT. Distant staging is usually managed by CT, with MR and FDG PET/CT which can be used as problem-solving techniques. Follow-up is performed according to the general recommendations of the oncological societies.

**Clinical relevance statement:**

It is essential to summarize each phase of colorectal cancer workup, differentiating the management for colon and rectal cancer supported by the main international guidelines and literature data, with the aim to inform the community on the best practice imaging in colorectal cancer.

**Key Points:**

*• Colorectal cancer is a prevalent disease that lends itself to imaging at each stage of detection and management.*

*• Various imaging modalities can be used as adjuncts to, or in place of, direct visualization methods of screening and are necessary for evaluating metastatic disease.*

*• Reevaluation of follow-up strategies should be considered depending on patients’ individual risk of recurrence.*

## Key recommendations

1. Colorectal cancer screening is mandatory to reduce incidence and mortality. Several recommended screening tests are available, including CT colonography (LoE HIGH, USPTSF recommendations).

2. Imaging is complementary to colonoscopy for diagnosis of CRC and mandatory for local and distant staging, restaging, and follow-up (LoE HIGH, ESMO guidelines).

3. Follow-up is performed according to the general recommendations of the oncological societies. However, a new personalized approach targeting high-risk patients should be pursued to improve patient adherence and to optimize costs, considering that an intensive follow-up in patients treated with a curative approach did not affect the survival (LoE HIGH, COLOFOL Trial).

## Introduction

Colorectal cancer (CRC) is a significant global health concern, being the third most diagnosed cancer and the fourth leading cause of cancer-related deaths worldwide. Sporadic CRC represents about 70% of all cases and only 5% are related to known hereditary conditions such as Lynch syndrome and familiar adenomatous polyposis. The remaining cases have apparent familial predisposition with no identifiable single germline mutations. An average-risk individual has an approximately 4 to 5% lifetime risk for developing colorectal cancer [1].

Early detection through screening, effective management through accurate diagnosis and staging, personalized treatment, and strict follow-up play a pivotal role in improving patient outcome. This is made apparent by observing the overall 5-year survival of a patient affected by CRC dropping from 93.6% if diagnosed at stage I to 13.4% at stage IV [2].

The areas in which efforts must be concentrated include the promotion of screening to increase adherence to screening programs, the improvement in quality of diagnostic tests, including for staging and for response to therapy, and enabling the development of precision medicine through individualized treatment plans.

This paper will review the current role of imaging in the different diagnostic steps of CRC.

## CRC screening

Colon carcinogenesis is a multistep process that involves stepwise accumulation of molecular and genetic defects in colonic epithelial cells, the formation of an adenoma, and eventually the transition to carcinoma. This process typically occurs over 10 to 15 years. The slowness of the process makes colon cancer a particularly favorable disease for screening. Studies have shown that early detection of CRC through screening significantly improves survival rates and reduces the need for aggressive treatments. Furthermore, identifying and removing benign precursors during screening can prevent the development of cancer altogether, thus reducing cancer incidence.

Currently, there are several options for CRC screening, each with its own performance characteristics and considerations for practice [3]. Available screening tests are grouped into “stool-based” and “visual” tests. “Stool-based” tests include fecal immunochemical test (FIT) and guaiac-based fecal occult blood test (gFOBT). “Visual tests” include flexible sigmoidoscopy (FS), colonoscopy (CC), and computed tomography colonography (CTC). They are targeted to the detection of cancers and polyps, thus able to reduce both mortality and incidence. Based on randomized clinical trials (RCTs) and model studies, biennial FIT/gFOBT, single and 5-yearly FS, and 10-yearly CC screening significantly reduce CRC-specific mortality, progressing from a reduction of 18% using gFOBT/FIT to a reduction of 70% using CC. The model estimates are much higher than in RCTs, because the simulated biennial gFOBT assumes higher adherence. Screening becomes more effective with younger initiation ages and increased adherence rates [4]. No direct data about reduction of CRC incidence and mortality are available for CTC. In comparative RCTs (CTC vs FIT, vs FS, and vs CC), CTC performance for the detection of advanced adenomas was better than stool tests, but slightly worse than CC, with an observed large adenoma detection rate of 5.4% for CTC and 6.3% for CC [5].

**Fig. 1:**
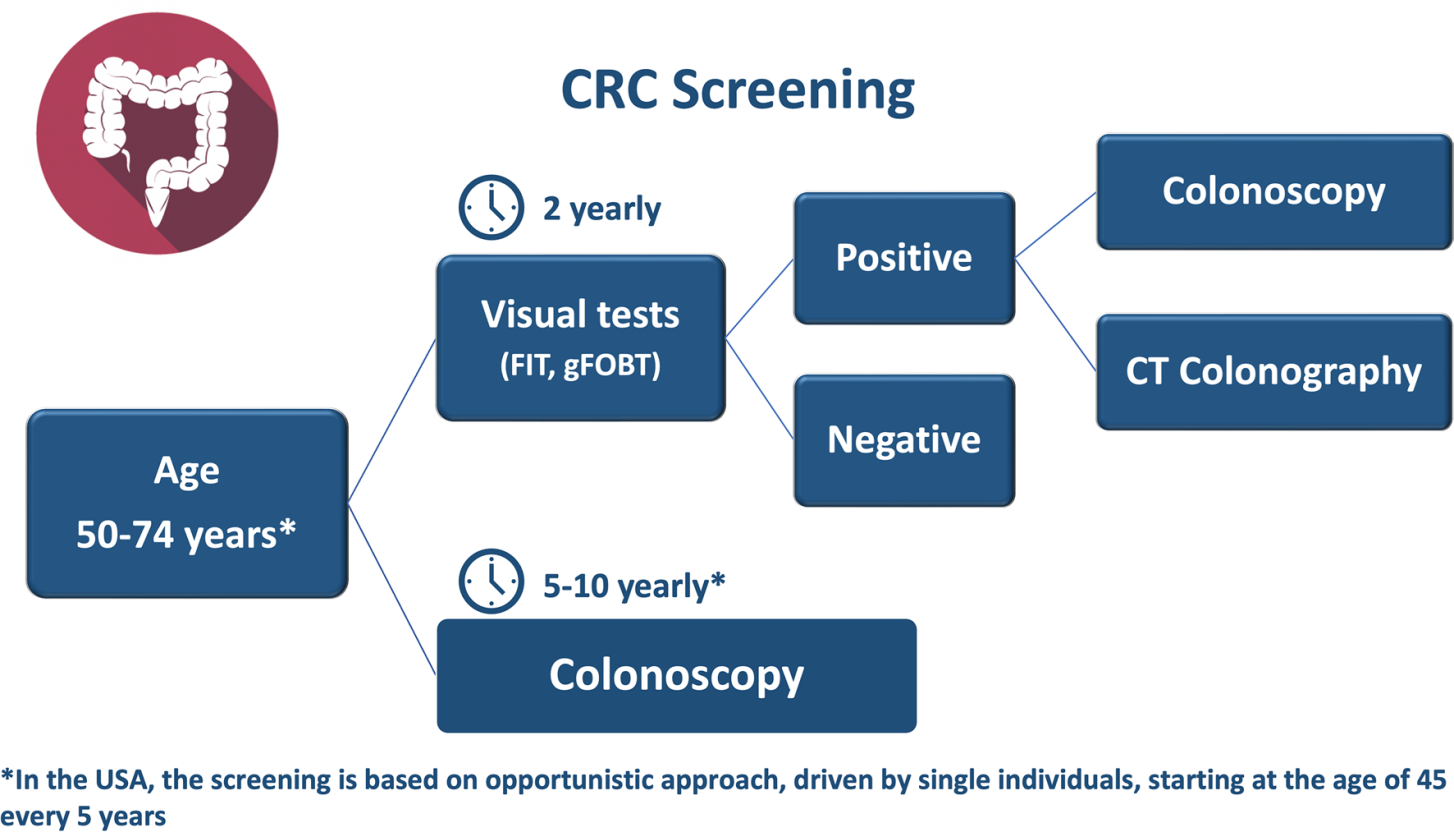
The flowchart of colorectal cancer screening. gFOBT, guaiac-based fecal occult blood test; FIT, fecal immunochemical test

The guidelines for CRC screening differ worldwide based on population risk, resources, and political approach. In Europe, most countries implement population-based programs, driven by national health authorities, inviting asymptomatic individuals, aged 50–75 years, to undergo a non-invasive test, usually FIT; two countries, Poland and Germany, advocate the use of CC. In organized screening programs, the role of CTC is limited to the study of individuals with a positive stool test unwilling or unable to undergo CC or in patients with an incomplete CC [6]. In the USA, an opportunistic approach, driven by single individuals, is preferred and the examinations recommended by the leading scientific societies (i.e., the American Cancer Society (ACS)) [7] and the United States Preventive Services Task Force (USPSTF) [8] are either a high sensitivity stool-based test or a visual test starting at the age of 45 (Fig. 1).

All positive outcomes on non-colonoscopy screening tests should be promptly followed up with a CC as part of the screening procedure. In case of negative result, time interval depends on the chosen screening test. However, in individuals with a family history of CRC or other risk factors, screening may begin earlier and require more frequent surveillance [6, 7].

## CRC diagnosis

CRC is most detected and diagnosed at CC performed in either a symptomatic patient or in a screening setting. Lesion biopsy is mandatory for patient management, not only to have histologic confirmation of the nature of the lesion, but also to assess cancer genetic profile [9].

In the case of incomplete CC or in fragile/elderly patients unable to undergo CC, CTC can be considered a valuable alternative for the diagnosis. CTC has also a pivotal role to exclude synchronous lesions in case of incomplete CC in order to plan the adequate surgical treatment [10].

Alternatively, CRC can be incidentally detected during a routine abdominal examination (CT or less commonly US, [^18^F]FDG PET/CT, or MRI) performed for a different diagnostic purpose, or at the emergency room in a patient presenting with symptoms of bowel occlusion/subocclusion or perforation [11].

Although CC is the gold standard for the detection of CRC and polyps, numerous studies have demonstrated a significant polyp miss rate and, more importantly, demonstrated the occurrence of interval cancers in the years after CC. Low operator skill and poor technology of the equipment were identified as main factors responsible for poor performances. This led to the development of indicators to ensure the quality of CC including among others bowel preparation, cecal intubation rate, withdrawal time, adenoma detection, and complication rate [12]. Similar studies introduced the concept of quality of CTC exams, intended as detection rate of CRC and high-risk colorectal polyps, that is strictly dependent on CTC technique (e.g., distension, CT protocol, and colonic preparation) and radiologists’ interpretation (e.g., CT software and training) [13].

## CRC staging

Staging is a crucial aspect of CRC management that guides treatment decisions and provides valuable prognostic information. The AJCC (TNM) staging system [14] allows healthcare providers to accurately assess the extent of the disease, determine the most appropriate treatment approach, and offer patients the best chance of successful outcomes. Imaging is critical in local and distant staging and to respond to oncologic requests. Different techniques are used for the rectum and colon.

## Local staging of colon cancer

Until recently, local staging of colon cancer has not been considered of major interest since the treatment of choice was represented by surgery. However, first results of a clinical trial [15] investigating potential advantages of neoadjuvant chemotherapy over standard postoperative chemotherapy for locally advanced colon cancer have demonstrated that this chemotherapy regimen can be delivered safely, without increasing perioperative morbidity, and produces marked histopathologic down-staging, fewer incomplete resections, and better 2-year disease control. As a result, there is increased interest for better radiologic criteria to identify high-risk stages. CT reports must include (1) evaluation of wall infiltration, identifying high-risk patients (cT3cd and cT4a); (2) assessment of tumoral involvement of extramural veins (EMVI) status; and (3) assessment of retroperitoneal margin for lesions located in ascending and descending colon. Although CT is the imaging modality of choice in local staging of colon cancer, some preliminary studies are advocating a potential role of MR, outperforming CT, similar to what happens in rectal cancer cases. Both modalities have clear limitations in characterizing pathologic lymph nodes (sens., 64–85% for CT and 64–93% for MR; spec., 71–95% for CT and 83–87% for MR) [16].

## Local staging of rectal cancer

**Fig. 2:**
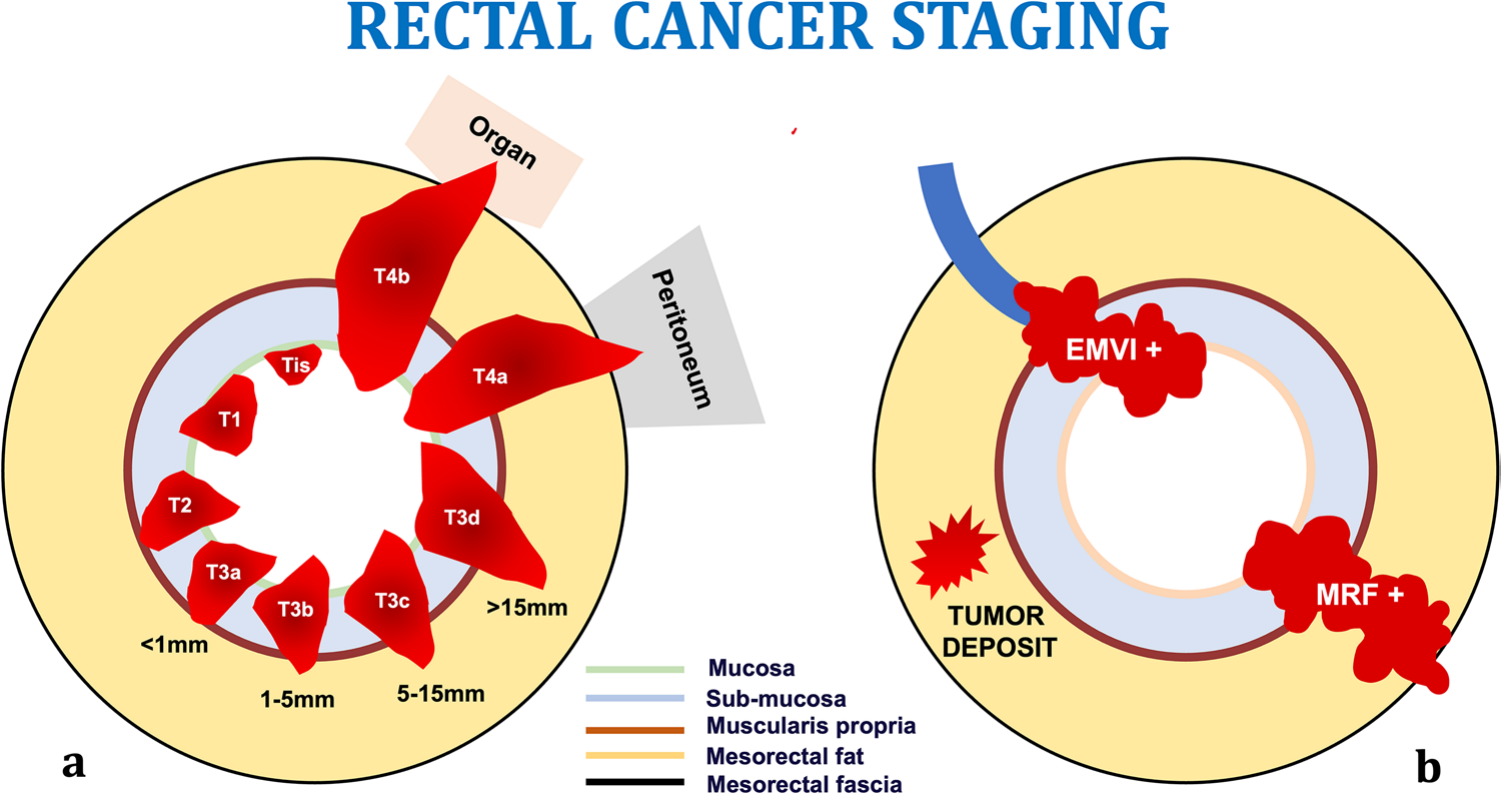
Rectal cancer local staging. T stage is represented in **a**, from Tis, limited to the mucosa, to T4b, involving other organs. In **b** are shown the most important high-risk factors, tumor deposit in the mesorectal fat, extramural venous invasion, and mesorectal fascia involvement, that are not influence on TNM staging

The role of imaging includes not only the assessment of wall infiltration (T-staging) and lymphadenopathies (N-staging), but also the detection of additional negative prognostic factors, e.g., status of mesorectal fascia (the so-called circumferential resection margin (CRM)), tumoral involvement of EMVI (Fig. 2), presence of tumor deposits, and infiltration of anal sphincters for low rectal cancers. These assessments make it possible to stratify patients into high-, intermediate-, and low-risk categories. This categorization allows for personalization of therapy, from upfront surgery in low-risk patients to different combinations of chemotherapy and short/long-course radiotherapy in intermediate- and high-risk patients, followed by different surgical options.

Endocavitary ultrasound is the imaging modality of choice for non-invasive rectal tumors (cT1–2) since high-resolution probes may discriminate different layers of the wall and provide useful information to refer patients either to mini-invasive surgery or to endoscopic resection. MRI is recommended for intermediate/advanced rectal cancers (cT3–T4) because of the higher accuracy in assessing extramural involvement and high-risk findings, such as EMVI, CRM status, and tumor deposits [17]. Assessment of pathologic lymph nodes is still an issue, despite the improvements offered by the combination of dimensional with morphologic criteria, yielding an accuracy of 64.2% and an AUC of 0.657 [18].

## Restaging/assessment of response to neoadjuvant therapy in rectal cancer

Assessment of cancer response after neoadjuvant chemoradiotherapy (CRT) has become crucial since now different personalized therapeutic options other than conventional surgery (i.e., organ-preserving surgery or wait-and-watch strategy) can be offered to patients with a complete response. Detailed radiological evaluation is mandatory, and MRI is the most accurate test. With MRI, the definition “near-complete” response is preferred. This refers to the absence of pathologic signal within fibrotic scar, which cannot completely rule out the presence of residual small clusters of vital neoplastic cells [19]. Best performances in assessing complete or near-complete response are guaranteed by a combination of MRI and endoscopy with an overall accuracy ranging from 80 to 92% [20].

MR image interpretation after neoadjuvant CRT represents a challenge for radiologists, with an overall accuracy of 64% in categorizing the response, sensitivity of 65%, and specificity of 63% [21]. No established criteria exist for T- and N-restaging. Different morphologic approaches have been proposed to classify tumor regression grade (TRG) (e.g., modified MRI, modified-mrTRG, and DWI-pattern), based on either T2-weighted or preferably a combination of T2-weighted and DWI images. Recently, the use of contrast-enhanced fat-saturated T1-weighted images has been advocated. The expertise of the radiologist is particularly important to improve MRI performance [22].

## Distant staging of rectal and colon cancer

CT is the workhorse for CRC distant staging, because of its availability, robustness, and reproducibility. However, limitations in detecting small liver lesions, peritoneal nodules, and bone metastases are well known [23]. MRI, using a combination of diffusion-weighted imaging and a hepatospecific contrast agent (i.e., Gd-EOB-DTPA), is the imaging modality of choice in patients referred to liver surgery [24]; consolidated data have demonstrated that this has the highest sensitivity among the non-invasive imaging methods, on both per-patient and per-lesion analysis. Regarding peritoneal carcinomatosis, both CT and MRI were comparable and good in assessing the peritoneal cancer index, one of the most important surgical prognostic scores [25], although more recent evidence slightly favors MR [26]. In selected cases, [^18^F]FDG PET/CT can be used to improve detection of distant metastases compared to CT. In the pre-operative clinical setting, [^18^F]FDG PET/CT has been shown to change the surgical management in 10.6% of patients [27]. New radiopharmaceutical agents (e.g., [^68^Ga]FAPI PET), with better performance detecting small metastatic liver, peritoneal, and brain lesions compared with [^18^F]FDG PET/CT, might further improve the role of PET imaging [28].

## CRC follow-up

CRC follow-up has the main goal of detecting cancer recurrence at an early stage, thereby maximizing patient survival in the metastatic setting. Intensive follow-up has been shown to increase the detection of recurrences; however, heterogeneity of the literature data does not allow the creation of unique algorithms. Since the vast majority (around 80%) of recurrences occur within the first 3 years and another 15% between the 3rd and the 5th year, a more intensive follow-up is recommended during the first 3 years and a stop after 5 years [29]. However, a recent trial demonstrated that repeated CT scans, every 6 months in the first 2 years from the surgery, in patients who received a curative treatment could not affect the survival [30]. These controversial results might suggest performing further investigations to deeply understand the real benefit of aggressive follow-up, and possibly to redesign the follow-up strategy, by stratifying patients in low-risk and high-risk categories with at least two different pathways.

Currently, apart from physical examination, CEA blood levels, and CC, the imaging modality of choice is CT of the chest, abdomen, and pelvis to be repeated every 6–12 months for the first 3 years in patients who are at higher risk of recurrence according to the TNM classification.

## Summary statements

Colorectal cancer (CRC) is the third most diagnosed cancer and the fourth leading cause of cancer-related deaths worldwide. Diagnostic imaging has a prevalent role in the evaluation of CRC from screening through follow-up. CRC screening is mandatory to reduce incidence and mortality. In a screening setting, CTC can be considered a support examination to CC or alternatively an upfront screening test. Although CC is the most common modality to diagnose CRC, CRC can be also incidentally detected during a routine abdominal imaging examination (including CT, CTC, or less commonly US and MR) or at the emergency department in patients presenting with intestinal occlusion/subocclusion or perforation. Once CRC is diagnosed, imaging is mandatory for local and distant staging. In local staging of rectal cancer, MR provides diagnostic and prognostic information useful to categorize patient risk and personalize patient treatment. Distant staging of CRC is managed through CT, MR, and FDG PET/CT used as problem-solving techniques (Table 1). Imaging is also critical in the assessment of response to therapy, both in neoadjuvant and adjuvant settings. Follow-up is performed according to the general recommendations of the oncological societies.

**Table 1:** Colorectal cancer workup from the screening to the follow-up

	**Tests**	**Notes**
**Screening**		**Reduction of mortality rate**
	Stool-based • Fecal immunochemical test • Guaiac-based fecal occult blood test	From 18 to 21% (2 yearly) [4]
	Visual-based • Flexible sigmoidoscopy • Colonoscopy • CT colonography	From 41 to 73% (5–10 yearly) [4]
**Diagnosis**		**Importance**
	Colonoscopy plus biopsy	Essential [9]
	CT	Incidental or emergency [9, 12]
	MR	Incidental [12]
**Staging**		**Type**
	Colon: • CT scans (chest, abdomen, and pelvis) • FDG PET/CT	Local and distant staging [9, 25] Distant staging [28]
	Rectum: • MR or endocavitary US • CT scans (chest, abdomen, and pelvis)	Local staging [9] Distant staging [9]
**Restaging**		**Type**
	Colon: • CT (chest, abdomen, and pelvis)	Local and distant restaging [9], [25]
	Rectum: • MR or endocavitary US • CT (chest, abdomen, and pelvis)	Local restaging [23, 24] Distant restaging [23, 24]
**Follow-up**		**Timing recommendation**
	• CEA blood test • CT (chest, abdomen, and pelvis) • Colonoscopy	Every 6–12 months for the first 5 years [9], [25]

## Patient summary

**Fig. 3:**
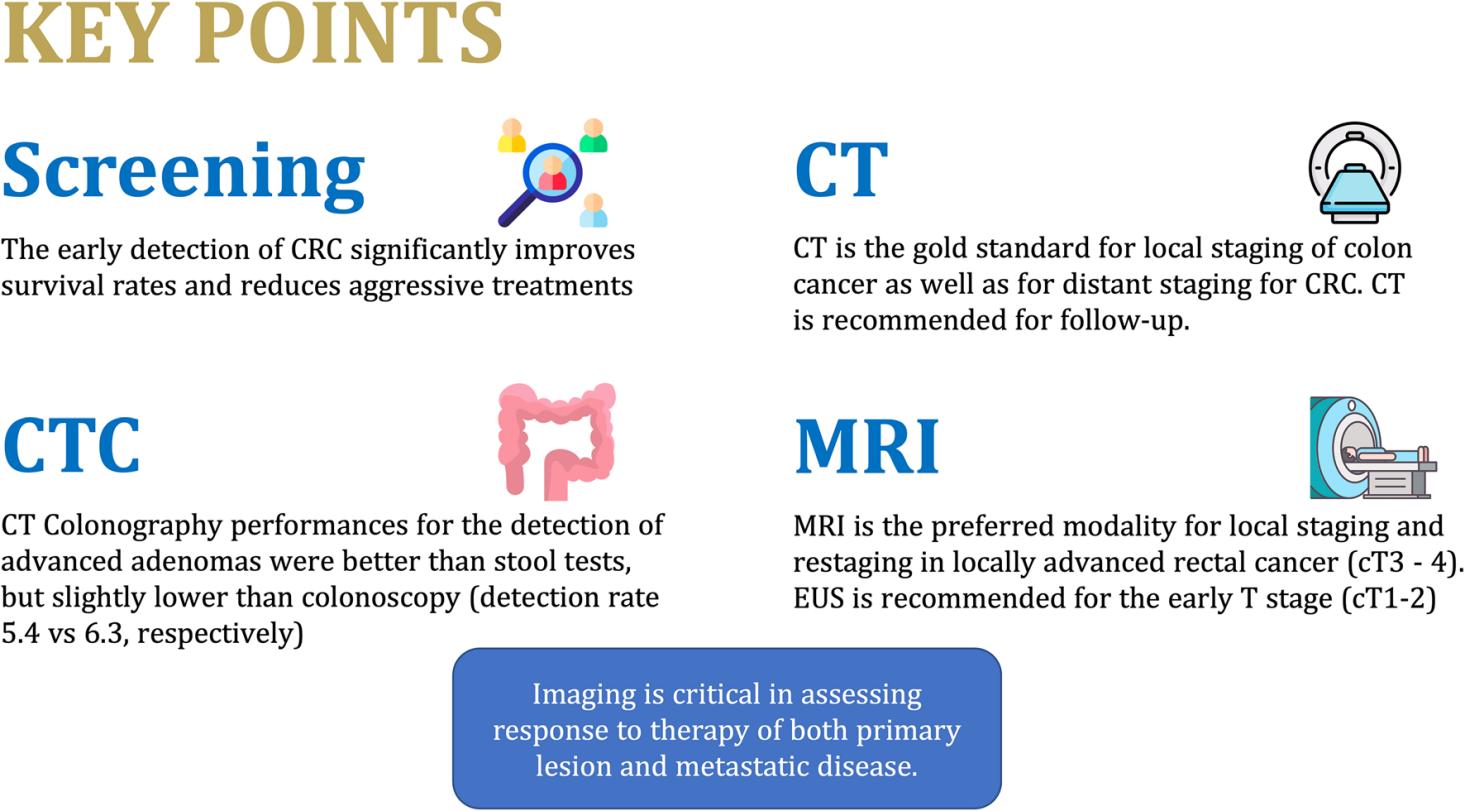
Key points regarding screening of colorectal cancer, the performance of CTC, and the advantages of CT and MRI

Colorectal cancer (CRC) is a significant global health concern, in both males and females. Early cancer detection and effective multidisciplinary management improve patient outcomes, and in each step, imaging is an important factor (Fig. 3). In CRC screening, CT colonography can be an alternative as an upfront screening test. Once CRC is diagnosed, multimodal imaging is mandatory for disease balance and to decide the most appropriate therapy (surgery and/or chemoradiotherapy); the choice of the most appropriate technique depends on cancer location and stage. Imaging is also critical in the assessment of response to therapy and in patient follow-up.

## Abbreviations

CC: Colonoscopy
CRC: Colorectal cancer
CRM: Circumferential resection margin
CTC: CT colonography
EMVI: Tumoral involvement of extramural veins
FIT: Fecal immunochemical test
FS: Flexible sigmoidoscopy
gFOBT: Guaiac-based fecal occult blood test
RCT: Randomized clinical trials

## Declarations

### Guarantor

The scientific guarantor of this publication is Prof. Andrea Laghi.

### Statistics and biometry

No complex statistical methods were necessary for this paper.

### Methodology

• review
